# Editorial: Computational Methods for Analysis of DNA Methylation Data

**DOI:** 10.3389/fbinf.2022.926066

**Published:** 2022-06-17

**Authors:** Pietro Di Lena, Christine Nardini, Matteo Pellegrini

**Affiliations:** ^1^ Department of Computer Science and Engineering, University of Bologna, Bologna, Italy; ^2^ CNR IAC “Mauro Picone”, Consiglio Nazionale delle Ricerche (CNR), Rome, Italy; ^3^ Department of Molecular, Cell and Developmental Biology, UCLA, Los Angeles, CA, United States

**Keywords:** DNA methylation, DNA methylation age, epigenetic clocks, copy number variations, cell-type deconvolution, methylation pattern imputation

DNA methylation is among the most studied epigenetic modifications in eukaryotes. The interest in DNA methylation stems from its role in development, as well as its well-established association with phenotypic changes. Particularly, there is strong evidence that methylation pattern alterations in mammals are linked to developmental disorders and cancer (Kulis and Esteller, 2010). Owing to its potential as a prognostic marker for preventive medicine, in recent years, the analysis of DNA methylation data has garnered interest in many different contexts of computational biology (Bock, 2012). As it typically happens with omic data, processing, analyzing and interpreting large-scale DNA methylation datasets requires computational methods and software tools that address multiple challenges. In the present Research Topic, we collected papers that tackle different aspects of computational approaches for the analysis of DNA methylation data. These manuscripts address novel computational solutions for copy number variation detection, cell-type deconvolution and methylation pattern imputation, while others discuss interpretations of well-established computational techniques.

Over the last 10 years, DNA methylation profiles have been successfully exploited to develop biomarkers of age, also referred to as epigenetic clocks (Bell et al., 2019). Epigenetic clocks accurately estimate both chronological and biological age from methylation levels. DNA methylation age and, most importantly, its deviation from chronological age have been shown to be associated with a variety of health issues. More recently, a second generation of epigenetic clocks has emerged. The new generation of clocks incorporates not only methylation profiles but also environmental variants, such as smoking and alcohol consumption, and they outperform the first generation in mortality prediction and prognosis of certain diseases. In our collection, the review by Chen et al. compares the first and second generation of epigenetic clocks that predict cancer risk and discusses pathways known to exhibit altered methylation in aging tissues and cancer.

Differentially methylated regions (DMRs), that is genomic regions that show significant differences in methylation levels across distinct biological and/or medical conditions (e.g., normal vs. disease), have been reported to be implicated in a variety of disorders (Rakyan et al., 2011). As a result, identifying DMRs is one of the most critical and fundamental challenges in deciphering disease mechanisms at the molecular level. Although DNA methylation patterns remain stable during normal somatic cell growth, alterations in genomic methylation may be caused by genetic alterations, or vice versa. However, standard DMR analysis often ignores whether methylation alterations should be viewed as a cause or an effect. Rhamani et al. discuss the effect of model directionality, i.e. whether the condition of interest (phenotype) may be affected by methylation or whether it may affect methylation, in differential methylation analyses at the cell-type level. They show that correctly accounting for model directionality has a significant impact on the ability to identify cell type specific differential methylation.

Different cell types exhibit DMRs at many genomic regions and such rich information can be exploited to infer underlying cell type proportions using deconvolution techniques. DNA methylation-based cell mixture deconvolution approaches can be classified into two main categories: reference-based and reference-free. While the latter are more broadly applicable, as they do not rely on the availability of methylation profiles from each of the purified cell types that compose a tissue of interest, they are also less precise. Reference-based approaches use DMRs specific to cell types (reference library) to determine the underlying cellular composition within a DNA methylation sample. The quality of the reference library has a big impact on the accuracy of reference-based approaches. Bell-Glenn et al. present RESET, a framework for reference library selection for deconvolution algorithms exploiting a modified version of the Dispersion Separability Criteria score, for the inference of the best DMRs composing the library, contributing to *de facto* standards (Koestler et al., 2016). In short, RESET does not require researchers to identify *a priori* the size of the reference library (number of DMRs), nor to rely on costly associated purified cells’ mDNA profiles.

Within a cellular population, the methylation patterns of different cell types and at specific genomic locations are indicative of cellular heterogeneity. Alterations of such heterogeneity are predictive of development as well as prognostic markers of diseases. Computational methods that exploit heterogeneity in methylation patterns are typically constrained by partially observed patterns due to the nature of shotgun sequencing, which frequently generates limited coverage for downstream analysis. One possible solution to overcome such limitations is offered by Chang et al. presenting BSImp, a probabilistic based imputation method that uses local information to impute partially observed methylation patterns. They show that using this approach they are able to recover heterogeneity estimates at 15% more regions with moderate sequencing depths. This should therefore improve our ability to study how methylation heterogeneity is associated with disease.

Finally, recent studies have shown how the associations between Copy Number Variations (CNVs) and methylation alterations offer a richer and hence more informative picture of the samples under study, in particular for tumor data characterized by large scale genomic rearrangements (Sun et al., 2018). Consequently, recent technological and methodological developments have enabled the possibility to measure CNVs from DNA methylation data. The main advantage of DNA methylation based CNV approaches is that they offer the possibility to integrate both genomic (copy number) and epigenomic (methylation) information. Mariani et al. propose MethylMasteR, an R software package that integrates DNA methylation-based CNV calling routines, facilitating standardization, comparison and customization of CNV analyses. This package, built into the Docker architecture to seamlessly mange dependencies, includes four of the most commonly used routines for this integrated analysis, ChAMP (Morris et al., 2014), SeSAMe (Zhou et al., 2018), Epicopy (Cho et al., 2019), plus a custom version of cnAnalysis450k (Knoll et al., 2017), overall enabling analysis of comparative results.

All the topics in this issue, although limited to specific aspects of DNA methylation analysis, highlight the importance of research in this field, the associated computational challenges and illustrate the significant impact that this type of data will likely have on preventive medicine.
